# Dynamics of high-sensitivity cardiac troponin T during therapy with balloon pulmonary angioplasty for chronic thromboembolic pulmonary hypertension

**DOI:** 10.1371/journal.pone.0204683

**Published:** 2018-09-25

**Authors:** Steffen D. Kriechbaum, Christoph B. Wiedenroth, Till Keller, Jan Sebastian Wolter, Ruth Ajnwojner, Karina Peters, Moritz A. Haas, Fritz C. Roller, Andreas Breithecker, Andreas J. Rieth, Stefan Guth, Andreas Rolf, Dirk Bandorski, Christian W. Hamm, Eckhard Mayer, Christoph Liebetrau

**Affiliations:** 1 Kerckhoff Heart and Thorax Center, Department of Cardiology, Bad Nauheim, German Center for Cardiovascular Research (DZHK), Partner Site Rhine-Main, Frankfurt am Main, Germany; 2 Kerckhoff Heart and Thorax Center, Department of Thoracic Surgery, Bad Nauheim, Germany; 3 Justus Liebig University Giessen, Department of Radiology, Giessen, Germany; 4 Gesundheitszentrum Wetterau, Department of Radiology, Bad Nauheim, Germany; 5 Justus Liebig University Giessen, Medical Clinic I, Division of Cardiology, Giessen, Germany; 6 Justus Liebig University of Giessen, Department of Internal Medicine, Division of Pulmonology, Giessen, Germany; Klinikum Region Hannover GmbH, GERMANY

## Abstract

**Aims:**

Balloon pulmonary angioplasty (BPA) is an interventional treatment modality for inoperable chronic thromboembolic pulmonary hypertension (CTEPH). Therapy monitoring, based on non-invasive biomarkers, is a clinical challenge. This post-hoc study aimed to assess dynamics of high-sensitivity cardiac troponin T (hs-cTnT) as a marker for myocardial damage and its relation to N-terminal pro-B-type natriuretic peptide (NT-proBNP) levels as a marker for cardiac wall stress.

**Methods and results:**

This study included 51 consecutive patients who underwent BPA treatment and completed a 6-month follow-up (6-MFU) between 3/2014 and 3/2017. Biomarker measurement was performed consecutively prior to each BPA and at 6-MFU.

In total, the 51 patients underwent an average of 5 BPA procedures. The 6-month survival rate was 96.1%. The baseline (BL) meanPAP (39.5±12.1mmHg) and PVR (515.8±219.2dyn×sec×cm^-5^) decreased significantly within the 6-MFU (meanPAP: 32.6±12.6mmHg, P<0.001; PVR: 396.9±182.6dyn×sec×cm^-5^, P<0.001). At BL, the median hs-cTnT level was 11 (IQR 6–16) ng/L and the median NT-proBNP level was 820 (IQR 153–1872) ng/L. The levels of both biomarkers decreased steadily after every BPA, showing the first significant difference after the first procedure. Within the 6-MFU, hs-cTnT levels (7 [IQR 5–12] ng/L; P<0.001) and NT-proBNP levels (159 [IQR 84–464] ng/l; P<0.001) continued to decrease. The hs-cTnT levels correlated with the PVR (r_rs_ = 0.42; p = 0.005), the meanPAP (r_rs_ = 0.32; p = 0.029) and the NT-proBNP (r_rs_ = 0.51; p<0.001) levels at BL.

**Conclusion:**

Non-invasive biomarker measurement provides valuable evidence for the decreasing impairment of myocardial function and structure during BPA therapy. Changes in hs-cTNT levels are suggestive for a reduction in ongoing myocardial damage.

## Introduction

Chronic thromboembolic pulmonary hypertension (CTEPH) occurs in about 0.1 to 9% of all patients surviving acute pulmonary embolism [1]. Due to a distinct impairment of pulmonary hemodynamics and secondary right heart dysfunction, the prognosis of CTEPH is poor without therapy. [2] Pulmonary endarterectomy is the treatment of choice, offering a potential curative approach. [1] For patients deemed to be inoperable, targeted medication and a consecutive balloon pulmonary angioplasty (BPA) as an interventional treatment option is recommended. [1, 3–7]

Risk prediction is essential for patient-centric care but therapy monitoring with non-invasive biomarker measurement is debatable as these option is considered to be inferior to invasive hemodynamic assessment and cardiac imaging. [1] However, since secondary impairment of cardiac function determines the prognosis in the progression of CTEPH, non-invasive detection of cardiac damage might be a valuable diagnostic adjunct.

Natriuretic peptides are shown to have a predictive value regarding therapy response and right ventricular recovery after pulmonary endarterectomy and BPA. [8–11] We could recently provide data about decreasing N-terminal pro-B-type natriuretic peptide levels (NT-proBNP) after BPA with the possibility of therapy monitoring and identification of patients who are BPA non-responders. [8] Cardiac troponins have diagnostic and prognostic value in various cardiovascular diseases. Cardiac troponins are used as outstanding markers for risk stratification and therapy guiding in acute coronary syndrome patients. [12] They serve further to redefine myocardial infarction, and risk stratification in patients with pulmonary embolism and have finally also become a risk factor in apparently healthy subjects. In addition, first data indicate the possibility of cardiovascular risk reduction mirrored by a decrease of high-sensitive troponin I due to blood pressure lowering in patients with arterial hypertension. [13]

The role of troponin in CTEPH patients undergoing BPA and its relation to NT-proBNP levels is not well described. It can be speculated that a decrease of pulmonary hemodynamics after BPA is accompanied by a decrease of troponin level, which might represent a cardiovascular risk reduction. Therefore, the aim of the present study was to characterize the time course of high-sensitively measured troponin T in patients undergoing BPA as a staged procedure and to determine relation to pulmonary hemodynamics and NT-proBNP levels in the periprocedural episode and at six months follow-up.

## Methods

The principles of the clinical and scientific work-up of patients who undergo treatment for CTEPH at our center have been recently published by our group. [8] The study population and the respective methods are described in brief as follows.

### Study population

This study included fifty-one consecutive patients undergoing BPA treatment at the Kerckhoff Heart and Thorax Center and completed a 6-month follow-up (6-MFU) after the final BPA treatment between March 2014 and March 2017. The performed pre- and post-procedural diagnostic and therapeutic work up of patients suffering from CTEPH was published by our group. [3, 14, 15] The routinely performed diagnostic work up of all patients includes clinical examination, 12-lead ECG, laboratory tests, 6-minute walk tests (6-MWD), echocardiography, CT angiography, right-heart catheterization, and pulmonary angiography. [3, 8] The findings of all patients were assessed in an interdisciplinary CTEPH conference to proof the final diagnosis of CTEPH in accordance with the current guidelines and to define the individual therapeutic concept. [1, 7] Primarily, the patients were evaluated regarding their technical operability with regards to the localization of the target lesions and the operability in dependence to the patients’ comorbidities. Consecutively the distinct staged BPA sequence was planned. In line with our standard clinical practice, the BPA procedures were performed by a dedicated BPA team (interventional radiologist, cardiologist, and thoracic surgeon). [3] The BPA sessions are performed with an interval of about 4 to 8 weeks. In preparation of each consecutive BPA procedure, the patients underwent follow-up examinations, adjusted to the individual requirements, but always including a re-evaluation of the clinical status and the laboratory findings. Six months after the completed BPA sequence, all patients underwent a comprehensive in-house follow-up examination, including a reassessment of clinical status, hemodynamics, cardiac function, laboratory findings, and functional capacity.

The investigation conforms with the principles outlined in the *Declaration of Helsinki*. All patients enrolled in the study gave written informed consent, which included consent for biomarker analyses. The study concept was approved by the ethics board of the Justus Liebig University of Giessen (AZ 43/14).

### Balloon pulmonary angioplasty and right heart catherization

BPA was performed as staged procedure under conscious sedation using femoral or jugular access as previously described. [3] Right heart catheterization (RHC) was performed as a part of the preprocedural diagnostic work-up and within the 6-MFU after the completed BPA sequence. [1] Usually a 6F sheath in the right internal jugular vein and a standard Swan-Ganz catheter were used for the RHC. To allow a reliable assessment of hemodynamics, as close to the real-life conditions as possible, we performed no modification of the given medication prior or during the RHC. In particular, no vasoactive agents were administered. [8]

### Laboratory assessment

At baseline (BL), prior to each BPA procedure and at the 6-MFU, venous blood samples for biomarker (hs-cTnT, NT-proBNP) were collected in plain tubes. The measurement of high-sensitivity cardiac troponin T (hs-cTnT) was performed with a high-sensitivity electro-chemiluminescence immunoassay (hs-cTnT assay, Elecsys Analyzer 2010, Roche Diagnostics, Mannheim, Germany). The limit of detection (LOD) is 5ng/l. Due to this LOD, we used 5ng/l as the lowest level of hs-cTnT in the statistical analysis. The limit of quantification is 13ng/l. The lowest level, measurable with a coefficient of variation (CV) <10%, is 13ng/l. The recommended cut-off value for ACS decision making with this assay is 14 ng/l. The measurement of NT-proBNP in serum used an electrochemiluminescence immunoassay with monoclonal antibodies (NT-proBNP assay, Elecsys Analyzer 2010, Roche Diagnostics, Mannheim, Germany). The LOD for this assay is 5.0 ng/l, whereas levels above the measuring range are reported as >35,000 ng/l. The lowest level measurable with a CV of 20% is 50.0 ng/l and at the cut-off value of 150 ng/l the CV is <3%. The upper limit of normal is 300.0 ng/l. [16]

### Statistical analysis

The results for continuous variables are displayed as mean ± standard deviation (SD) or as median and interquartile range (IQR), as appropriate. Categorical variables are expressed as the absolute number and the percentage of the whole cohort. Parametric distribution was assessed using the Shapiro-Wilk test. Subcohorts at BL and 6-MFU were compared with the Student t-test for normally distributed parameters and the Mann-Whitney-U test for all other continuous variables. Dynamics of parameters that were obtained at baseline and at the 6-MFU underwent paired sample testing with the Student’s t-test for normally distributed parameters and the Wilcoxon signed-rank test for all other continuous variables. Bivariate parametric Pearson’s correlations were analyzed for selected clinical and hemodynamic parameters as well as laboratory findings. All statistical tests were performed with SPSS software, version 19.0. A two-tailed *P* value <0.05 was considered to be statistically significant.

## Results

### Clinical characteristics and periprocedural data

Baseline characteristics of the evaluated 51 patients (28 women; mean age [±SD] 63.1±11.5 y) are summarized in Table 1. In all patients, the indication for BPA therapy was a technically inoperable status with peripheral target lesions in 47 (92.2%) patients and a status after PEA with recurrent pulmonary hypertension in 4 patients (7.8%). All patients in our cohort were on oral anticoagulation therapy for >3 months and in 29 (56.9%) patients a specific medical treatment for pulmonary hypertension was established. In total 265 (mean 5/patient) BPA interventions with a treatment of 410 (mean 8/patient) vessels were performed. The most frequent complications after BPA were hemoptysis in 7.4% and reperfusion injury in 3.4% of all interventions. The survival rate in the 6-MFU was 96.1%.

**Table 1:** Sociodemographic characteristics, comorbidities, and medication at baseline.

Parameter	N or Mean (±SD) or Median (IQR)	%
Age at 1^st^ BPA, y	63.1 (±11.5)	
Female gender	28	54.9
Body-mass index, kg/m^2^	25.7 (±3.8)	
Current smoker	14	27.5
Diabetes mellitus	5	9.8
Dyslipidemia	7	13.7
Arterial hypertension	31	60.8
Chronic renal failure	10	19.6
GFR, ml/min	79.3 (62.2–93.9)	
Creatinine, μmol/l	0.94 (0.78–1.13)	
Atrial fibrillation	3	5.9
History of stroke	5	9.8
Coronary artery disease	9	17.9
History of cancer	9	17.6
Chronic obstructive pulmonary disease	4	7.8
History of acute pulmonary embolism	23	45.1
History of deep vein thrombosis VT	6	11.8
Procoagulant coagulopathy	2	3.9
OAC	51	100
ERA	6	11.8
PDE5 inhibitor	7	13.7
Riociguat	21	41.2
Riociguat alone	17	33.3
PDE5 inhibitor alone	5	9.8
ERA alone	2	3.9
Riociguat + PDE5 inhibitor	1	2.0
Riociguat + ERA	3	5.9
PDE5 inhibitor + ERA	1	2.0

Abbreviations: BPA = Balloon pulmonary angioplasty, ERA = endothelin receptor antagonist, GFR = glomerular filtration rate, OAC = oral anticoagulative therapy, PDE5 = phosphodiesterase type 5;

### Impact of BPA therapy on physical capacity and pulmonary hemodynamics

At baseline, 49 (96.1%) patients were in WHO functional class ≥III which decreased to 6 (11.8%) patients at the 6-MFU (P<0.001) (Table 2). The median 6-minute-walk distance increased significantly (375.0 m [IQR 281–446] at BL vs. 409 m [IQR 332–446] at the 6-MFU; P = 0.017). Table 2 presents the data of the RHC and echocardiographic measurements at baseline and at 6-MFU. The meanPAP (BL: 39.5±12.1 mmHg vs. 6-MFU: 32.6±12.6 mmHg; P<0.001) and the PVR (BL: 516±219 dyn×sec×cm^-5^ vs. 6-MFU: 397±183 dyn×sec×cm^-5^; P<0.001) decreased significantly.

**Table 2:** Functional, biomarker, echocardiographic, and hemodynamic data at BL and 6-MFU.

Parameter	Baseline	6-MFU	p-value
LVEF, %	60 (60–60)	65 (60–65)	0.002
TAPSE, mm	19 (13–20.5)	21.5 (17–24)	0.09
6-MWD, m	375.0 (281–445.5)	408.5 (332.3–445.8)	0.017
WHO FC (I-IV)	I:0; II:2; III:31; IV:18;	I:20; II:23; III:5; IV:1;	<0.001
Hs-cTnT, ng/l	11 (6–16)	7 (5–12)	<0.001
Hs-cTnT reduction, %	11 (0.0–43.0)		
NT-proBNP, ng/l	820.55 (153–1871.5)	159.3 (84.4–464.3)	<0.001
NT-proBNP reduction, %	53.6 (22.4–85.5)		
GFR, ml/min	79.3 (62.2–93.9)	79.6 (67.1–95.0)	0.22
Creatinine, μmol/l	0.94 (0.78–1.13)	0.88 (0.76–1.04)	0.09
RA pressure, mmHg	7.5 (±4.1)	6.1 (±2.7)	0.008
PCWP, mmHg	9.0 (8–12)	10.0 (8–11)	0.269
Diastolic PAP, mmHg	22.1(±8.2)	16.9 (±7.7)	<0.001
Systolic PAP, mmHg	67.8 (±21.6)	55.8 (±22.7)	<0.001
MeanPAP, mmHg	39.5(±12.1)	32.6 (±12.6)	<0.001
MeanPAP reduction, %	19.2 (4.3–28.7)		
PVR, dyn×sec×cm^-5^	515.8 (±219.2)	396.9(±182.6)	<0.001
PVR, reduction %	23.4 (4.4–34.7)		
SVO2, %	66.4 (61.5–70)	70.4 (76.5–73)	0.003
CI, l/min/m^2^	2.5 (±0.6)	2.5 (±0.5)	0.326

Abbreviations: CI = cardiac index, FC = functional class, LVEF = left ventricular ejection fraction, NT-proBNP = N-terminal pro-B-type natriuretic peptide, PAP = pulmonary artery pressure, PCWP = Pulmonary capillary wedge pressure, PVR = pulmonary vascular resistance, RA = right atrium, TAPSE = Tricuspid Annular Plane Systolic Excursion, WHO = World health organization, 6-MWD = 6-minute-walk-test-distance, 6-MFU = 6-month follow-up;

### Biomarkers at baseline and impact of BPA treatment on biomarker levels

At BL, the median hs-cTnT level was 11 (IQR 6–16) ng/L and the median NT-proBNP level was 820 (IQR 153–1872) ng/L. Among all patients, 16 (31.4%) showed a hs-cTnT above the 99^th^ percentile at baseline.

NT-proBNP as marker reflecting hemodynamic changes showed a robust reduction after BPA treatment with 821 ng/l at BL to 159ng/l at the 6-MFU as published recently. [8]

**Fig 1:**
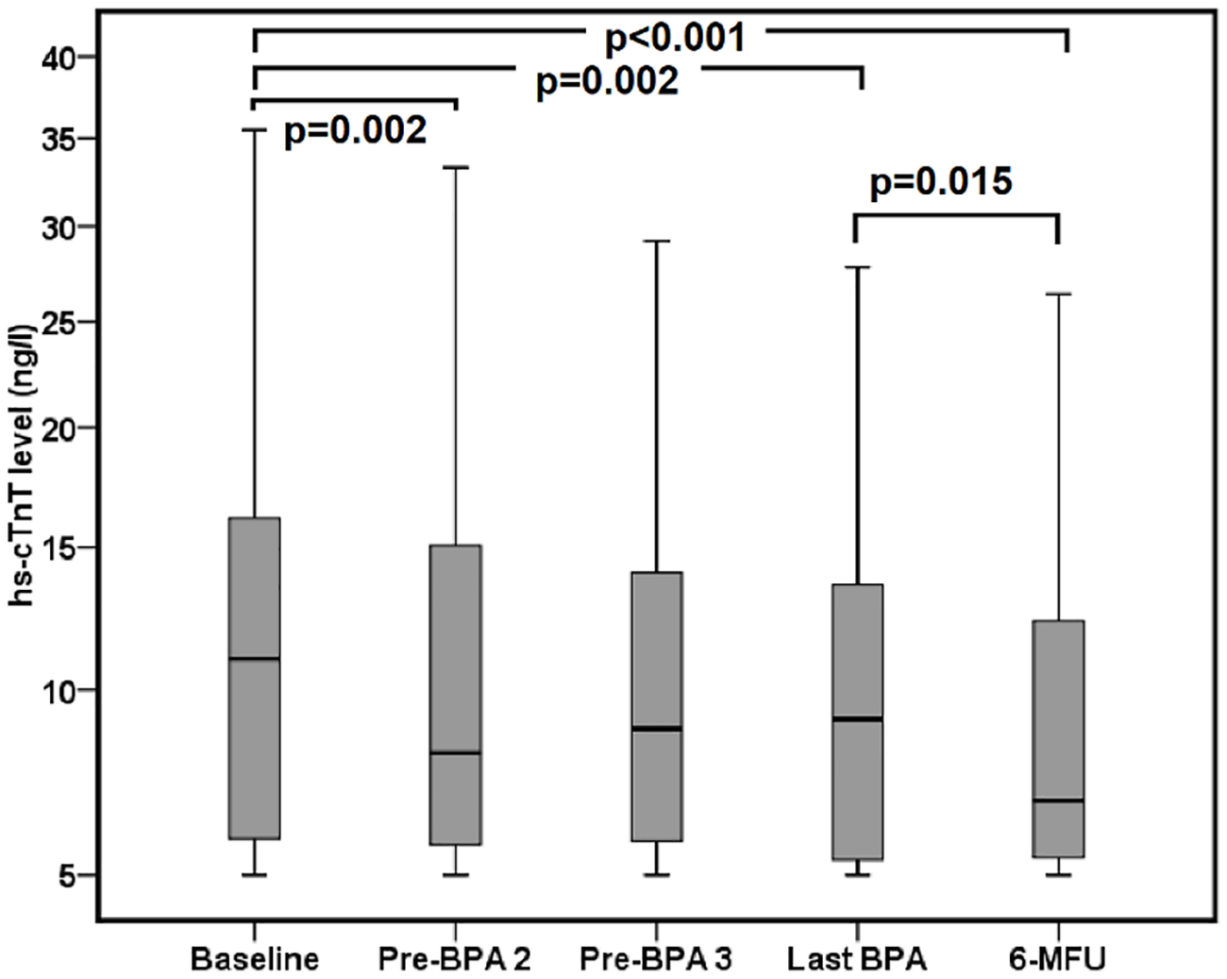
Analysis of the time course of hs-cTnT levels during staged BPA procedure (BPA = Balloon pulmonary angioplasty, hs-cTnT = high-sensitivity cardiac troponin T, 6-MFU = 6-month follow-up).

Comparison of serum hs-cTnT levels with BL values revealed a significant decrease at all pre-specified time points following the first BPA, with the lowest value being measured at the 6-MFU (11 ng/l [IQR 6–16] vs. 7 ng/l [IQR 5–12]; P<0.001) (Fig 1).

The median percentage change in hs-cTnT at the 6-MFU was a reduction of 11% (IQR 0% change to a decrease of 43%; range of percent change [min-max]: increase of 39.0 to a decrease of 86.0%; range of absolute change [min-max]: increase of 10 to a decrease of 50 ng/l) (Table 2).

Out of 51 patients, 16 (31.4%) showed unchanged (n = 8; 15.7%) or even increased (n = 8; 15.7%) hs-cTnT level at the 6-MFU. In 9 (17.6%) patients, the hs-cTnT level remained above the 99^th^ percentile at the 6-MFU. The 8 patients with unchanged hs-cTnT level were characterized by a static hs-cTnT level at the LOD (5ng/l) or below. Patients with an increase of the hs-cTnT level did not differ from the rest of the cohort regarding their functional baseline characteristics (age (p = 0.69), body mass index (p = 0.77), LV-EF (p = 0.43), TAPSE (p = 0.21), meanPAP (p = 0.63), PVR (p = 0.38), WHO-FC (p = 0.40), 6-MWD (p = 0.14), GFR (p = 0.45), serum creatinine level (p = 0.41).

### Association of hs-troponin T with the procedural extent, pulmonary hemodynamics, NT-proBNP levels and clinical outcome under BPA treatment

The hs-cTnT levels and the NT-proBNP levels correlated significantly (BL: r_rs_ = 0.51; p≤0.001; 6-MFU: r_rs_ = 0.42; p = 0.002). Correspondingly, patients with a persistent hs-cTnT level above the 99^th^ percentile at the 6-MFU were characterized by significantly (p<0.001) higher NT-proBNP levels.

Invasively determined meanPAP and PVR significantly correlated with hs-cTnT with r_rs_ = 0.32 (p = 0.029) and r_rs_ = 0.42 (p = 0.005) at BL. There was no significant correlation with the meanPAP (r_rs_ = 0.16; p = 0.27) and the PVR (r_rs_ = 0.26; p = 0.10) at 6-MFU.

The hs-cTnT levels did not correlate with the WHO FC at BL (r_rs_ = 0.24; p = 0.10) but at the 6-MFU: r_rs_ = 0.46; p = 0.001). The relative change of the baseline hs-cTnT did not correlate significantly with the number of treated vessels (r_rs_ = 0.11; p = 0.53) or the number of BPA sessions (r_rs_ = 0.25; p = 0.10).

Two patients died within the 6-MFU. In both deceased patients, an increase of the baseline hs-cTnT level was observed after the completion of the BPA sequence (patient 1: BL: 5.6 ng/l vs. last BPA: 8.4ng/l; patient 2: BL: 16.7ng/l vs. last BPA: 20.8ng/l).

### Baseline hs-troponin T to stratify patients before BPA therapy

Among patients with a BL hs-cTnT above the 99^th^ percentile (16 patients, 31.4%), 14 patients (87.5%) showed a decrease of hs-cTnT level at 6-MFU. In this subgroup, 12 patients (75.0%) showed concomitant a significant decrease of NT-proBNP levels >25%.

NT-proBNP levels were significantly higher among patients with a BL hs-cTnT level above the 99^th^ percentile at BL (267.9ng/l [IQR 107.15–1212.5] vs. 1819.5ng/l [IQR 1112.0–4458.0]; p = 0.001) and within the 6-MFU (127.2ng/l [IQR 69.7–248.0] vs. 356.9ng/l [IQR 144–2010.0]; p = 0.005). Besides the NT-proBNP levels, the 16 patients with a BL hs-cTnT above the 99^th^ percentile, showed a significantly higher PVR (522 dyn×sec×cm^-5^ [IQR 339–668] vs. 328 dyn×sec×cm^-5^ [IQR 208–491]; p = 0.021)) and borderline significant higher meanPAP (35mmHg [IQR 29–47 vs. 27mmHg [IQR 23–36]; p = 0.65), lower 6-MWD (275m [IQR 327–431] vs. 429m [IQR 371–447]; p = 0.077) at the 6-MFU. The differences regarding the TAPSE (17mm [IQR 13–23] vs. 24mm [IQR 18–26]; p = 0.171), LVEF (63% [IQR 58–65] vs. 65% [IQR 61–65]; p = 0.296) were not statistically significant.

## Discussion

BPA is a promising treatment option for inoperable CTEPH patients. [1] Over the last decade, data about the beneficial effects accumulated and procedural improvements led to a high level of periprocedural safety. [3, 5, 6, 11, 14, 17, 18] In CTEPH, intravascular thrombotic obstruction compounded by vascular remodeling leads to an increased PVR and meanPAP. [1, 19, 20] The pathological changes of pulmonary hemodynamics trigger an impairment of cardiac, particularly right ventricular, function. [21, 22] Right ventricular afterload elevation causes increased wall tension and leads to myofibrillar damage. [23] Natriuretic peptide levels correlate with myocardial wall stress and proved to indicate right ventricular remodeling and cardiac troponins are highly sensitive for the detection of myocardial injury. [9, 24–26] Accordingly, the aim of the present study was to characterize the time course of hs-cTnT mirroring myocardial damage in CTEPH patients undergoing BPA and to determine the relation to NT-proBNP levels as an indicator for cardiac wall stress.

The main findings of this study are: 1) Hs-cTnT levels decrease substantially after BPA showing significant difference already after the first procedure; 2) The hs-cTnT decrease is most distinct in patients with a hs-cTnT level above the 99^th^ percentile at baseline. 3) The hs-cTnT levels correlate with NT-proBNP levels at baseline and in the follow-up indicating a relation to wall stretch induced hs-cTnT release.

Cardiac troponins I and T are the leading biomarkers for the detection of myocardial injury and are one corner stone in the diagnostic work up of suggested acute myocardial infarction. [12] However, cardiac troponins are not only released due to acute myocardial infarction. [23] Since the implementation of high-sensitive cardiac troponin assays with improved sensitivity, elevated cardiac troponin levels are regularly seen in patients with various cardiac diseases but no acute myocardial infarction. [23] Reversible conditions (cytosolic membrane leakage, transient ischemia, wall stretch) versus definite necrosis of cardiomyocytes are controversially discussed as underlying release mechanisms. [23, 27]

In our study, hs-cTnT levels were measured at BL and immediately in advance to each consecutive BPA session. Thus, the particular hs-cTnT level mirrors the degree of persistent myocardial stress followed by myocardial injury after an interval of 4 to 8 weeks to the previous BPA procedure.

Hs-cTnT levels decreased significantly starting with the first BPA treatment. It has to be mentioned, that NT-proBNP levels also decreased from BL to the last BPA but stabilized over time with no significant level changes after the last BPA compared to the 6-MFU. The hs-cTnT levels continuously decreased during all pre-specified time points including the 6-MFU. This observation indicates that the reverse cardiac remodeling process with decrease of right ventricular after load and therefore less ventricular wall stress starts already after the first BPA session. Interestingly, the reverse remodeling process seems to be ongoing beyond the last BPA procedure as indicated by further lowering hs-cTnT levels at the 6 months follow-up.

High-sensitivity cardiac troponin (hs-cTn) assays in daily clinical practice allow the assessment of low troponin levels with precise analytical accuracy. Cardiac troponin indicated disease severity and predicted worse outcome in mixed cohorts of patients with pulmonary hypertension. [28, 29] Völkers et al. observed elevated hs-cTnT levels in PH patients at rest and significant dynamics after the performance of cardiopulmonary exercise testing.

The hs-cTnT decrease was most pronounced in those patients who had hs-cTnT level above the 99^th^ percentile at baseline. NT-proBNP levels were significantly higher at BL and within the 6-MFU among those patients with a hs-cTnT level above the 99^th^ percentile at the 6-MFU.

NT-proBNP levels were significantly higher at the 6-MFU among those patients with a persistent hs-cTnT level above the 99^th^ percentile. Patients with a BL hs-cTnT above the 99^th^ percentile further showed higher PVR and meanPAP values and a lower 6-MWD at the 6-MFU. We hypothesize that hs-cTnT levels above the 99^th^ percentile at BL or a lack of decrease under therapy accompanied by elevated NT-proBNP levels indicate the disease severity and probably ongoing cardiac damage.

In this context Kimura et al. reported that patients with higher meanPAP, PVR, and BNP levels at BL to be those with the highest decrease of hs-cTnT levels in the follow-up.

In facts, it is not known up to which degree of right ventricular remodeling, the RV-dysfunction is reversible under BPA therapy. [30] Although the right ventricular origin of this release is not proven, hs-cTnT dynamics correlated significantly with meanPAP and NT-proBNP. [27] In this context, Andreassen et al. reported low levels of NT-proBNP and troponin T to be a an indicator for reduced right ventricular strain in CTEPH patients. [11]

To the best of our knowledge, this is the first study employing hs-cTnT measurement at every stage in CTEPH patients undergoing BPA as a staged procedure. Our results show that hs-cTnT is decreasing stepwise under therapy, indicating a decrease of ongoing myocardial damage presumably due to reduced right ventricular afterload after BPA therapy. This assumption is strengthened by correlation of hs-cTnT-levels and NT-proBNP levels at baseline and in the 6-MFU.

Our study indicates that consecutive hs-cTnT and NT-proBNP measurement under staged BPA therapy might help to assess the effects of BPA on hemodynamics and impairment of right ventricular structure and function, which would lead to a better monitoring of BPA therapy.

Some limitations of this study need to be mentioned. The study included a relatively small number of patients. Nevertheless, our BPA program is among the largest worldwide and the observed results clearly demonstrate the significant decrease in hs-cTnT levels from baseline at every stage of the procedure. High-sensitive troponin assays enabled the detection of lower protein levels, but we can still not definitely define the exact pathophysiological meaning of low-level cardiac troponin. The prognostic value of elevated hs-cTnT levels, even below the 99^th^ percentile has been investigated in in several non-CTEPH cohorts. The results of a large meta-analysis, including more than 65.000 individuals of a general population, associated elevated troponin concentrations, also below the 99^th^ percentile, with a higher rate of mortality. [31] The assessment of the exact diagnostic value of low-level cardiac troponins in CTEPH patients undergoing BPA therapy requires prolonged follow-up periods. At present we can state, that in our cohort, even patients with troponin below the 99^th^ percentile showed significant reduction of their baseline levels, which suggests to be response to reduced cardiac wall stress.

Cardiac troponin levels might also be influenced by other conditions like heart failure worsening or other adverse cardiac events. Within the follow-up we detected no progression of coronary artery disease or myocardial infarction within the 9 patients suffering from coronary artery disease at baseline. Left ventricular heart failure worsening seems to be unlikely in our cohort in face of a slight improvement of LVEF under BPA therapy.

In conclusion hs-cTnT is elevated in CTEPH patients and indicates ongoing subclinical myocardial damage presumably triggered by increased right ventricular afterload. The hs-cTnT level decreases significantly under BPA therapy and correlates with the reduction of right ventricular wall stress, indicated by NT-proBNP levels.
